# Supplementary material for: Vacancy-mediated anomalous phononic and electronic transport in defective half-Heusler ZrNiBi
Source: Nat Commun. 2023 Aug 5;14:4722. doi: 10.1038/s41467-023-40492-7 (PMC10404254; doi:10.1038/s41467-023-40492-7)
Supplement: Supplementary file 1 — Supplementary Information [file 41467_2023_40492_MOESM1_ESM.pdf]

Supplemental Material for "Vacancy-mediated anomalous phononic and electronic transport in defective half-Heusler ZrNiBi"

Wuyang Ren[1,2,†], Wenhua Xue[3,†], Shuping Guo[4,†], Ran He[4], Liangzi Deng[2], Shaowei Song[2], Andrei Sotnikov[4], Kornelius Nielsch[4], Jeroen van den Brink[4], Guanhui Gao[5], Shuo Chen[2], Yimo Han[5], Jiang Wu[1], Ching-Wu Chu[2], Zhiming Wang[1,*], Yumei Wang[3,*], Zhifeng Ren[2,*]

[1] Institute of Fundamental and Frontier Sciences, University of Electronic Science and Technology of China, Chengdu 610054, People's Republic of China
[2] Department of Physics and Texas Center for Superconductivity at the University of Houston (TcSUH), Houston, Texas 77204, United States
[3] Beijing National Laboratory for Condensed Matter Physics, Institute of Physics, Chinese Academy of Science, Beijing 100190, People's Republic of China
[4] Leibniz Institute for Solid State and Materials Research, Dresden 01069, Germany
[5] Department of Materials Science and Nano-Engineering, Rice University, Houston, Texas 77005, United States

† These authors contributed equally to this work.

* Corresponding Authors: zhmwang@uestc.edu.cn; wangym@iphy.ac.cn; zren@uh.edu.

Single parabolic band model

According to the single parabolic band (SPB) model, the Hall carrier concentration (n_H) and the Seebeck coefficient (S) are given by[1]

$$n_H = \frac{4\pi(2m^* k_B T)^{3/2}}{h^3} \frac{4F_0^2(\xi_F)}{3F_{-1/2}(\xi_F)} \tag{S1}$$

$$S = -\frac{k_B}{e}\left[\frac{2F_1(\xi_F)}{F_0(\xi_F)} - \xi_F\right] \tag{S2}$$

$$F_n(\xi_F) = \int_0^\infty \frac{\chi^n}{1+\exp(\chi-\xi_F)} d\chi \tag{S3}$$

$$\xi_F = \frac{E_F}{k_B T} \tag{S4}$$

where h is the Planck constant, k_B is the Boltzmann constant, e is the electron charge, m^* is the density of states effective mass, $F_n(\xi_F)$ is the Fermi integral, ξ_F is the reduced Fermi energy, and χ is the reduced carrier energy.

Deduction for $(\bar{M})^{-1/2}$ dependence of v_s

The relationship between sound velocity (v_s) and the average atomic mass in a unit cell ($\bar{M}$) can be deduced by interconnecting kinetic and elastic energy as follows[2]

$$v_s = [\frac{1}{3}(\frac{2}{v_t^3} + \frac{1}{v_l^3})]^{-1/3} \quad \text{(S5)}$$

$$v_t = (\frac{G}{\rho})^{1/2} \quad \text{(S6)}$$

$$v_l = (\frac{4G+3B}{3\rho})^{1/2} \quad \text{(S7)}$$

$$\rho = \frac{n\bar{M}}{a^3 N_A} \quad \text{(S8)}$$

where v_t is the transversal sound velocity, v_l is the longitudinal sound velocity, G is the shear modulus, B is the bulk modulus, ρ is the density, n is the number of atoms in a unit cell, a is the lattice constant, and N_A is the Avogadro constant. Following Equations S5-S8, v_s shows a $(\bar{M})^{-1/2}$ dependence:

$$v_s = (\bar{M})^{-1/2}(\frac{a^3 N_A}{n})^{1/2}(ELA)^{-1/3} \quad \text{(S9)}$$

$$ELA = \frac{2}{3}(G)^{-3/2} + \frac{1}{3}(\frac{4}{3}G + B)^{-3/2} \quad \text{(S10)}$$

By comparing the sound velocity of possible ternary half-Heuslers from the *AFLOWLIB.org* database[3] (denoted as v_s^{DB}) and that deduced from Equations S9 and S10 (denoted as v_s^{ELA}), the deviation is within ~3%. The corresponding data are shown in Table S1.

Table S1. Elastic property and sound velocity of possible ternary half-Heuslers from the *AFLOWLIB.org* database.

Material	Space group	Lattice constant a (Å)	Shear modulus G (GPa)	Bulk modulus B (GPa)	Sound velocity v_s (m s^{-1})	
					v_s^{DB}	v_s^{ELA}
ScNiSb	$F\bar{4}3m$	6.16	61.92	98.84	3403	3448
TiNiSn	$F\bar{4}3m$	6.00	60.94	134.2	3253	3314
TiCoSb	$F\bar{4}3m$	5.89	75.95	143.2	3499	3560
HfPdSn	$F\bar{4}3m$	6.37	53.74	122.7	2493	2545
TaRuSb	$F\bar{4}3m$	6.19	72.16	176	2769	2829
NbFeSb	$F\bar{4}3m$	5.95	79.44	166.8	3342	3406
ZrCoBi	$F\bar{4}3m$	6.19	62.85	122.6	2736	2786
NbRuSb	$F\bar{4}3m$	6.19	68.38	169.4	3046	3115
ScNiBi	$F\bar{4}3m$	6.30	50.3	86.94	2698	2735
YNiSb	$F\bar{4}3m$	6.35	54.64	87.63	3062	3102
YNiBi	$F\bar{4}3m$	6.46	49.22	83.67	2592	2629
TiPtSn	$F\bar{4}3m$	6.28	49.05	132.1	2465	2523
ScPdSb	$F\bar{4}3m$	6.41	41.98	96.36	2708	2761
ScPtSb	$F\bar{4}3m$	6.42	51.05	110	2595	2648
YPtSb	$F\bar{4}3m$	6.60	46.5	99.17	2440	2487
VFeSb	$F\bar{4}3m$	5.79	62.44	155.4	3099	3165
VRuSb	$F\bar{4}3m$	6.05	50.04	158.6	2705	2778

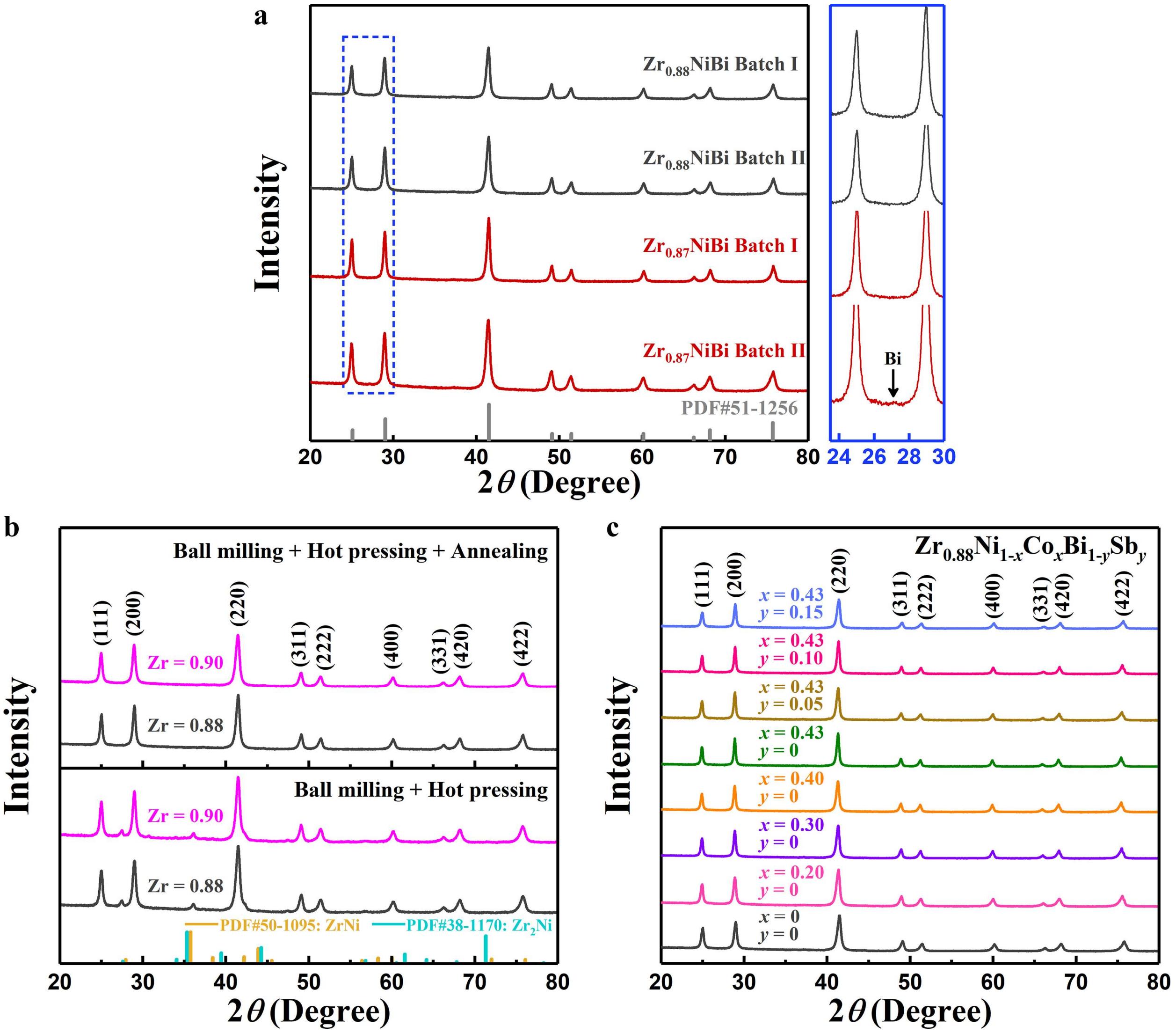

Figure S1. Structure characterization of ZrNiBi-based specimens. (**a**) XRD patterns for $Zr_{0.88}NiBi$ and $Zr_{0.87}NiBi$ from different batches synthesized using the same method. Right panel: magnified view between the (111) and (200) peaks. (**b**) XRD patterns for $Zr_{0.88}NiBi$ and $Zr_{0.90}NiBi$ with and without an additional annealing process. (**c**) XRD patterns for $Zr_{0.88}Ni_{1-x}Co_xBi_{1-y}Sb_y$.

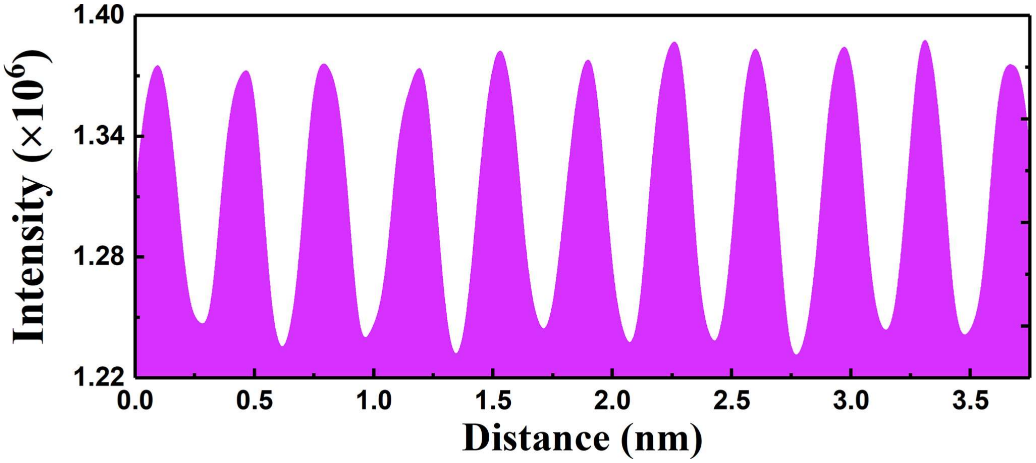

Figure S2. HAADF-STEM intensity profile within the Bi-atom plane of $Zr_{0.88}NiBi$.

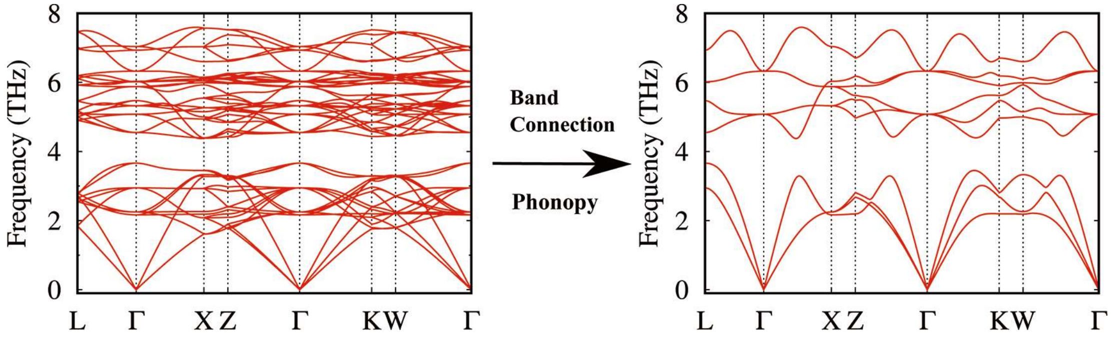

Figure S3. Phonon dispersions of $Zr_8Co_8Bi_8$ without and with estimations of band connections from eigenvectors in the Phonopy package.

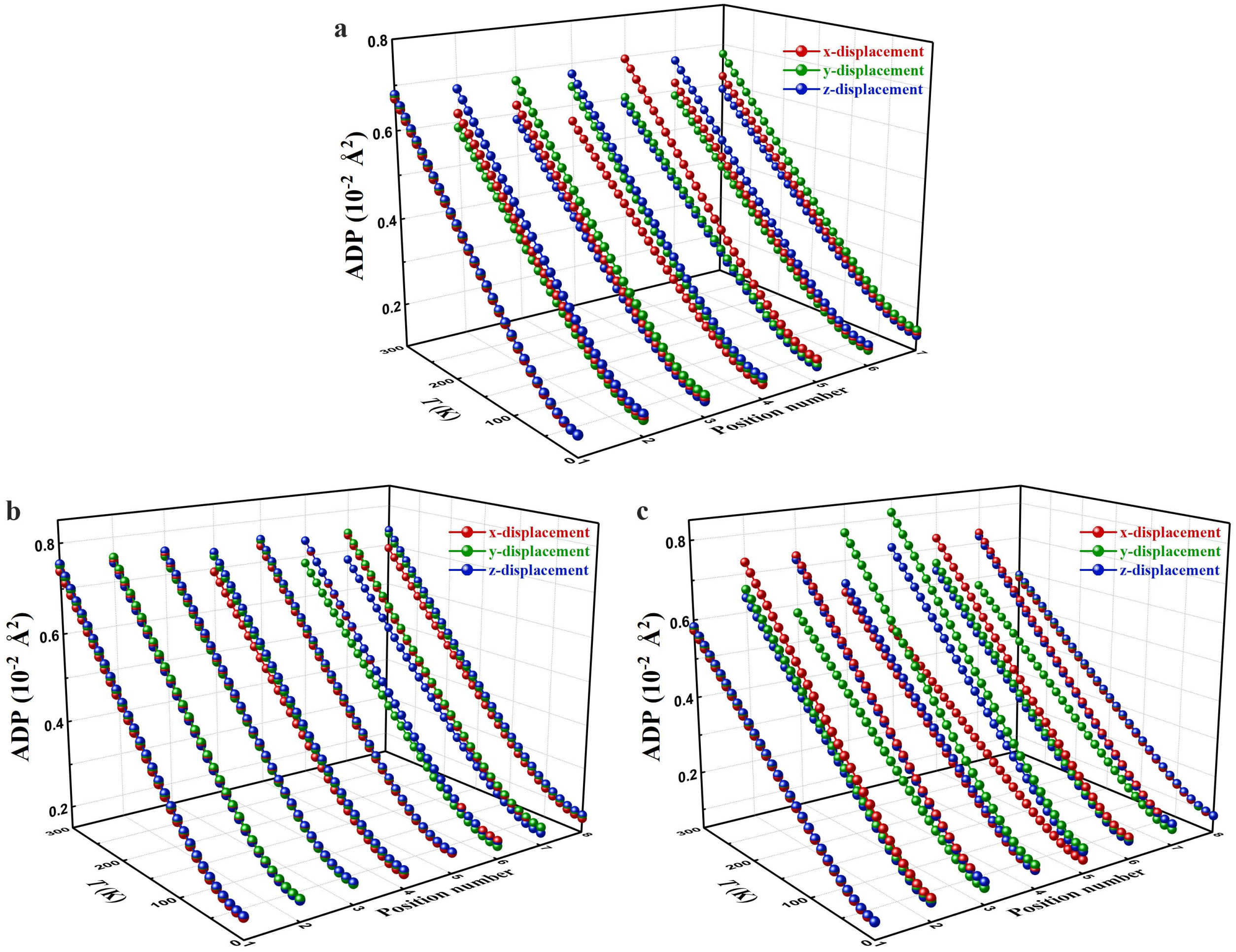

Figure S4. Atomic displacement parameters (ADPs) along the three Cartesian directions for (**a**) Zr, (**b**) Ni, and (**c**) Bi in the supercell of $Zr_{0.88}NiBi$.

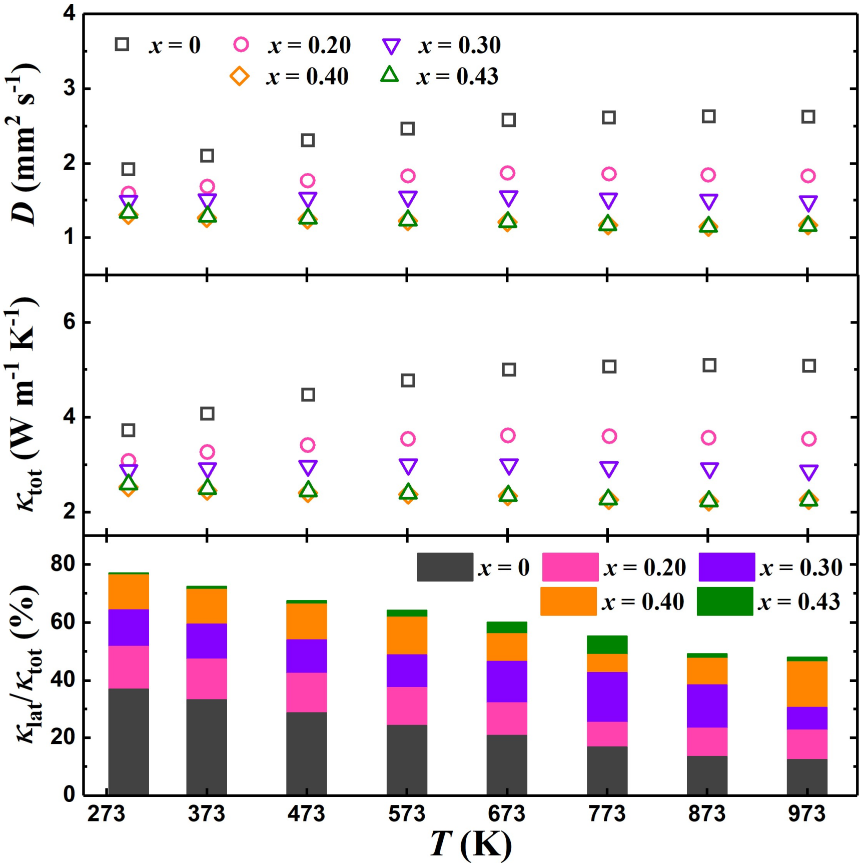

Figure S5. Thermal diffusivity (top), total thermal conductivity (middle), and lattice contribution to total thermal conductivity (bottom) for $Zr_{0.88}Ni_{1-x}Co_xBi$ at different temperatures.

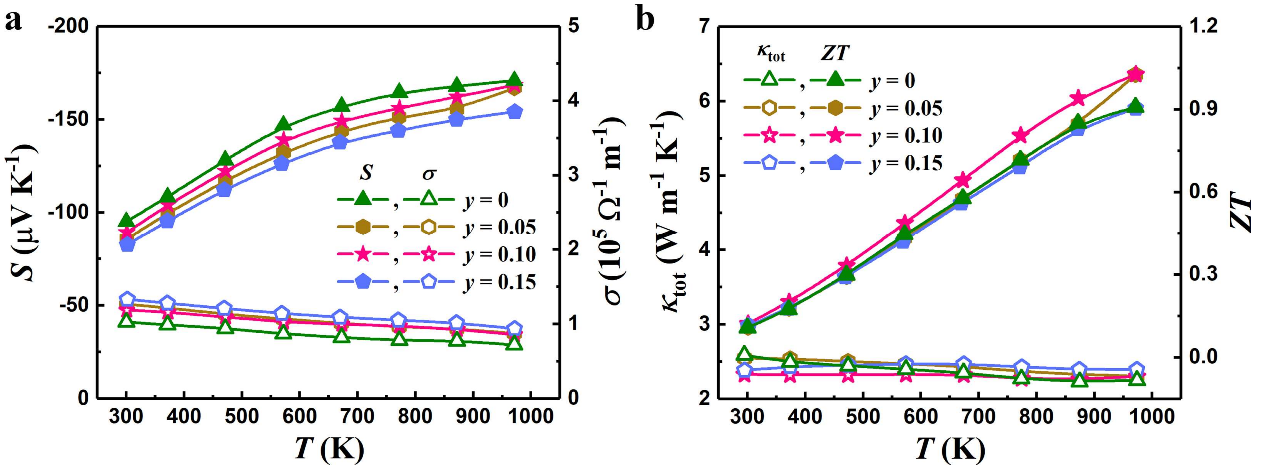

Figure S6. Temperature-dependent (**a**) Seebeck coefficient and electrical conductivity and (**b**) total thermal conductivity and figure-of-merit for $Zr_{0.88}Ni_{0.57}Co_{0.43}Bi_{1-y}Sb_y$.

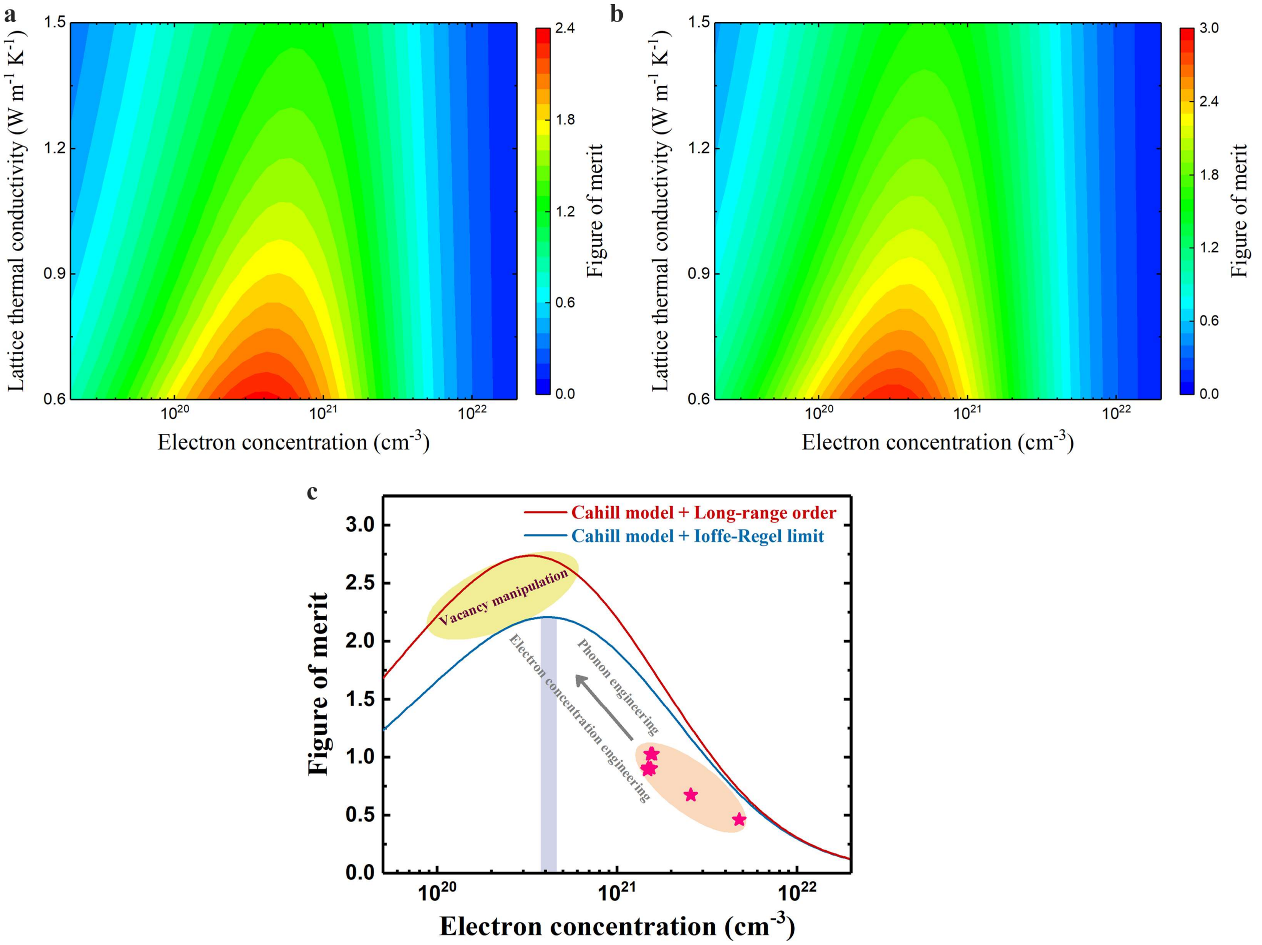

Figure S7. Modeled figure of merit for defective ZrNiBi at 973 K. Dependence of figure of merit on lattice thermal conductivity and electron concentration based on (**a**) the Ioffe–Regel condition and the Cahill model and (**b**) the long-range ordering condition and the Cahill model. (**c**) Comparison between experimentally determined performance (symbols) and modeled performance (solid lines). Gray bar: electron concentration range for figure of merit of ~2.2 determined by the Ioffe–Regel limit and the Cahill model.

References

1. He, R. et al. Achieving high power factor and output power density in p-type half-Heuslers $Nb_{1-x}Ti_xFeSb$. *Proc. Natl. Acad. Sci. USA* **113,** 13576-13581 (2016).
2. Rogl, G. et al. Mechanical properties of half-Heusler alloys. *Acta Mater.* **107,** 178-195 (2016).
3 Curtarolo, S. et al. AFLOW: an automatic framework for high-throughput materials discovery. *Comput. Mater. Sci.* **58**, 218-226 (2012).
